# Living-donor liver transplantation following cardiopulmonary bypass: A case report

**DOI:** 10.1097/MD.0000000000017230

**Published:** 2019-09-20

**Authors:** Min Seok Oh, Jeong Min Sung, Hyo Jin Yeon, Hyung Jun Cho, Justin Sangwook Ko, Gaab Soo Kim, Hyunyoung Lim

**Affiliations:** a Department of Anesthesiology and Pain Medicine, Samsung Medical Center, Sungkyunkwan University School of Medicine; b Department of Anesthesiology and Pain Medicine, Hanyang University Medical Center, Hanyang University College of Medicine, Seoul, Korea.

**Keywords:** cardiopulmonary bypass, end-stage liver disease, liver transplantation

## Abstract

**Rationale:**

Liver transplantation is an increasingly common treatment for patients with liver cirrhosis or hepatocellular carcinoma. Liver transplantation in patients with heart disease can pose a significant challenge to the transplant teams.

**Patient concerns:**

A 46-year-old woman was diagnosed with hepatitis B virus-related hepatocellular carcinoma 3 years ago and had received 3 times transarterial chemoembolization.

**Diagnoses:**

The patient was diagnosed as end-stage liver disease due to hepatocellular carcinoma and was scheduled to undergo living-donor liver transplantation. The preoperative echocardiogram revealed mass in the right atrium and the inferior vena cava.

**Interventions:**

The patient underwent mass removal under cardiopulmonary bypass followed by liver transplantation.

**Outcomes:**

A month later, she was discharged without any complications.

**Lessons:**

There have only been a few reported cases of anesthetic liver transplantation after a cardiopulmonary bypass. The successful experience described in this case report suggests that some patients may be eligible to undergo a liver transplantation after a cardiopulmonary bypass.

## 1 Introduction

Liver transplantation (LT) is an increasingly common treatment for patients with liver cirrhosis or hepatocellular carcinoma. End-stage liver disease (ESLD) itself is a major risk factor in general surgery. Patients with ESLD generally have various coagulopathy such as impaired synthesis of clotting factors, excessive fibrinolysis, etc.^[1]^ The cardiopulmonary bypass (CPB) is used in most cardiac surgeries and associated with platelet and coagulation defects, inflammation, and increased fibrinolysis, similar to the coagulopathies of ESLD.^[2]^ Therefore, LT in patients with heart disease can pose a significant challenge to the transplant teams because of the deterioration of these coagulation disorders.^[3]^ In particular, there have been no reports on the proper dose of heparin for treating patients with ESLD who are scheduled to have CPB. This case report is about this team's experience of living-donor LT after the removal of a mass in the right atrium (RA) and inferior vena cava (IVC) under CPB.

## 2 Case report

A 46-year-old female patient (weight: 54.3 kg, height: 155.2 cm) was admitted to undergo a living-donor LT. The written informed consent for patient information and images to be published was provided by the patient. She was diagnosed with hepatitis B virus-related hepatocellular carcinoma 3 years ago and had received 3 times transarterial chemoembolization. She had no underlying diseases and had a cesarean section 12 years ago. She was diagnosed as having 2 small nodules in the lower lobe of her right lung 2 years ago. They were found to be benign by a biopsy conducted during the preoperative workup.

A transthoracic echocardiogram revealed a mass in her RA and IVC. The size of the mass was 9 mm × 16 mm. Thoracic surgeons consented to remove the mass under CPB followed by LT given the results of the LT team's preevaluation of the patient. Although coagulopathy was a possibility due to the CPB, the CPB was thought to be tolerable because the patient's coagulation test results were within normal limits. Her platelet count was 119,000/μL, her prothrombin time (international normalized ratio) was 1.1, her activated prothrombin time was 32.7 seconds, and her fibrinogen level was 329 mg/dL. Her preoperative Child–Turcotte–Pugh (CTP) classification was grade A and her model for ESLD (http://en.wikipedia.org/wiki/Model_for_End-Stage_Liver_DiseaseMELD) score was 7.

**Figure 1:**
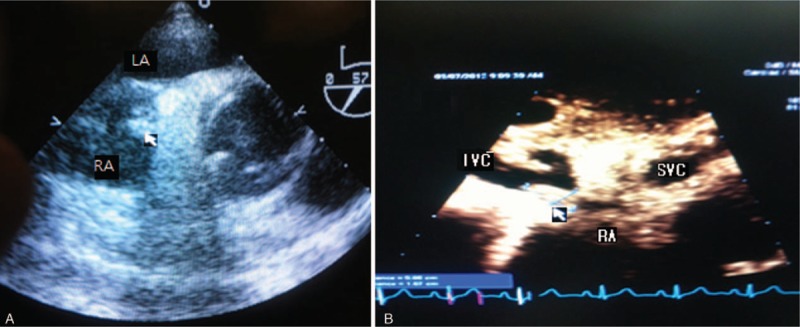
Transesophageal echocardiography finding. (A) In the midesophageal short axis view, the arrow indicates significant mass in RA. (B) In the bicaval view, the arrow indicates significant mass in IVC and RA. IVC = inferior vena cava, LA = left atrium, RA = right atrium, SVC = superior vena cava.

The patient was subject to 5-lead electrocardiography, pulse oxymetry, and noninvasive blood pressure monitoring in the operating room. Induction was carried out with 275 mg of pentothal and 8 mg of vecuronium and general anesthesia was maintained with sevoflurane in an oxygen-medical air mixture. The right radial artery, femoral artery, femoral vein, and internal jugular vein were subject to arterial and venous cannulation. A multifunction pulmonary artery catheter was inserted through a 9-Fr Advanced Venous Access catheter in the internal jugular vein but was placed at a depth of 15 cm due to the mass in the RA and IVC. A Fluid Management System (Belmont Instrument Corporation, Billerica, MA) was used because massive bleeding was expected. A transesophageal echocardiography probe was carefully inserted and produced results similar to the preoperative results (Fig. 1).

The operation began with an upper abdominal incision. The inhalation agent was changed from sevoflurane to isoflurane after an abdominal retractor was applied. There was no ascites. The cell saver, a blood suctional device, was applied. Plasma solution A was chosen as a main fluid and its infusion rate was adjusted by central venous pressure and pulse pressure variation. After the lever surgeon dissected the ligaments and the IVC around the liver, the thoracic surgeon performed a median sternotomy. After injecting 160 mg of heparin, activated clotting time (ACT) was over 1500 seconds. After the cannulation of aorta and superior vena cava, partial CPB of 50% was initiated. Dopamine was then continuously infused at a rate of 5 μg/kg/min right after partial CPB began due to decreased blood pressure. After a few minutes, the anhepatic phase began. IVC cannulation was done on the distal IVC away from mass and total CPB was started. After applying a cardioplegia solution, the RA and IVC were incised. Mass was extended from IVC wall to RA wall. En-bloc resection of the mass was impossible, so the IVC-RA junction and distal IVC were resected. The IVC was reconstructed using a bovine pericardium. The patient was weaned from CPB and vascular cannulations were removed. Norepinephrine was infused at a rate of 0.05 μg/kg/min to facilitate CPB weaning and was increased to 0.1 μg/kg/min to maintain blood pressure. Total CPB time was 81 minutes. ACT was 195 seconds after 150 mg of protamine was injected slowly. Before CPB weaning, 4300 mL of crystalloid, 300 mL of albumin, and 1500 mL of colloid were infused via CPB. Urine output was 520 mL.

The living-donor LT proceeded smoothly. After reperfusion, 30 μg of epinephrine was injected because systolic blood pressure dropped to approximately 65 mm Hg. After a few minutes, all vital signs stabilized. After reperfusion, 1000 mg of tranexamic acid was injected to reduce oozing at the operation site.

The operation took 14 hours. Throughout the procedure, the patient received 14,100 mL of plasma solution A, 500 mL of 6% hetastarch, 2000 mL of 6% tetrastarch, 200 mL of 5% dextrose, 760 mL 20% albumin, 12 units of leukocyte-depleted red blood cell, 6 units of fresh frozen plasma, 8 units of cryoprecipitate, and 1 unit of platelet pheresis. A total of 1688 mL of cell saver blood was collected and reinfused into the patient after filtering. Estimated intraoperative blood loss expressed as lost red cell mass^[4]^ was 3310 mL and total urine output was 5750 mL. Intraoperative hemodynamic and laboratory data, including thromboelastography results, are summarized in Table 1.

**Table 1:**
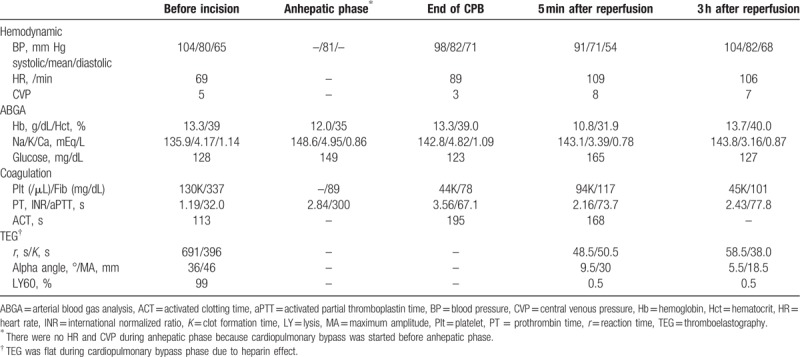
Intraoperative hemodynamic and laboratory data.

The patient was weaned from mechanical ventilation at postoperative day (POD) 2. Dopamine and norepinephrine infusions were stopped on POD 3. She was transferred from the intensive care unit to the general ward on POD 9. Her PT, aPTT, bilirubin, aspartate transaminase, and alanine transaminase laboratory test results normalized. No residual mass or anastomosis strictures were discovered by a postoperative echocardiogram. An abdominal color Doppler showed patent hepatic vasculature. She was discharged on POD 29. After 1 month, she has been examined in the outpatient clinic and found to not suffer any complications.

## 3 Discussion

There have been no reports on the proper dose of heparin for treating patients with ESLD who are scheduled to have CPB. In this case, we used 160 mg (16,000 IU) of heparin during CPB. It is recommended that the initial pre-CPB dosage of heparin for anticoagulation is 300 units/kg and that an ACT of <300 seconds is unsafe for CPB. An ACT of over 400 seconds prevents fibrin monomer appearance during CPB.^[5]^ In this case, the patient was given the recommended dose of heparin because her preoperative coagulation test results were within normal limits, but her ACT was 1500 seconds. There have only been a few reports about how to use heparin for CPB in patients with ESLD. Kujovich^[1]^ reported that 400 IU/kg of heparin was injected in CTP grade C resulting the maintenance of an ACT over 500 seconds with additional heparin injections as required. DeStephano et al^[6]^ reported that a median dose of heparin of 27,000 IU administered to nine patients (model for end-stage liver disease [MELD] score range: 11–24, body mass index range: 24.8–33.3, body weights not disclosed) result in ACTs longer than 480 seconds. Filsoufi et al^[7]^ administered 300 IU/kg of heparin to patients with liver cirrhosis (MELD score mean: 14.2, MELD score range: 7–29) undergoing heart surgery with CPB. The patient in this case study was incrementally treated with a heparin dose response curve. Lower doses of heparin may result in more thrombin generation, which in turn can render platelets hemostatically dysfunctional, thereby creating a bleeding risk following CPB.^[2]^ Further studies are required to determine the appropriate dosage of heparin for CPB in patients with ESLD.

The LT following CPB can increase the risk of massive bleeding beyond the heparin risk alone. ESLD itself is a major risk factor in general surgery. Patients with ESLD generally manifest impaired synthesis of clotting factors, excessive fibrinolysis, disseminated intravascular coagulation, thrombocytopenia due to impaired synthesis of thrombopoietin and immune destruction, and platelet dysfunction owing to circulating platelet inhibitors and deficiency of platelet glycoprotein receptors.^[1]^ The anhepatic phase during LT can induce more coagulopathy. Sabate et al^[8]^ reported that during the anhepatic phase, coagulopathy resulted from decreased clotting factors caused by surgical bleeding which is often aggravated by increased portal hypertension and the esophageal-gastric venous distension caused by compressive maneuvers and vascular clamping. Liver dysfunction is correlated with morbidity and mortality. Major postoperative complications were more frequent in CTP classes B and C than class A.^[7,9]^ In this case, the patient had a CTP class A and was deemed to be suitable for undergoing CPB. If her liver dysfunction was sufficiently severe, then an operation conducted in stages should have been more strongly considered. CPB is associated with platelet and coagulation defects, inflammation, and increased fibrinolysis, similar to the coagulopathies of ESLD.^[2]^ Furthermore, CPB is known to affect hemostasis by releasing numerous vasoactive substances and cytotoxic chemicals that affect coagulation, vascular resistance, vascular permeability, fluid balance, and major organ function. Additionally, hypothermia, hemodilution, and hypoperfusion during CPB may also be responsible for hemostatic compromise.^[10]^ In this regard, when combined with CPB, severe ESLD coagulopathies can result in massive intraoperative bleeding.

Some transplant centers have used antifibrinolytics to reduce intraoperative bleeding as they are effective in reducing red blood cell requirements.^[11]^ Massad et al^[12]^ and Axelrod et al^[13]^ used intravenous loading doses of 2,000,000 KIU of aprotinin followed by an infusion of 500,000 KIU/h during a combined coronary bypass and LT. Aprotinin seems to be more effective at controlling massive bleeding than other agents but also has a higher mortality risk. In 2007, it was withdrawn from the market because of safety concerns.^[14,15]^ Thus, in this case, 1000 mg of tranexamic acid, bolus, was administered after the reperfusion.

In conclusion, LT following CPB can be a safe and effective surgical option for patients with ESLD and heart problems. However, careful review of patient circumstances is necessary to increase the effectiveness of surgical outcomes. Coagulopathy and the clinical long-term outcomes of the combined procedures have yet to be evaluated.

## Author contributions

**Conceptualization:** Min Seok Oh, Justin Sangwook Ko, Gaab Soo Kim, Hyunyoung Lim.

**Data curation:** Min Seok Oh.

**Formal analysis:** Jeong Min Sung, Justin Sangwook Ko.

**Methodology:** Jeong Min Sung, Hyo Jin Yeon, Hyung Jun Cho.

**Resources:** Hyung Jun Cho.

**Software:** Hyo Jin Yeon, Hyung Jun Cho, Justin Sangwook Ko.

**Supervision:** Gaab Soo Kim.

**Writing – original draft:** Min Seok Oh.

**Writing – review & editing:** Gaab Soo Kim, Hyunyoung Lim.

Hyunyoung Lim orcid: 0000-0003-0343-6750.
